# Preparation and Characterization of Gelatin Nanofibers Containing Silver Nanoparticles

**DOI:** 10.3390/ijms15046857

**Published:** 2014-04-22

**Authors:** Lim Jeong, Won Ho Park

**Affiliations:** Department of Advanced Organic Materials and Textile System Engineering, Chungnam National University, Daejeon 305-764, Korea; E-Mail: ionvirus@hanmail.net

**Keywords:** Ag nanoparticles, gelatin nanofibers, electrospinning

## Abstract

Ag nanoparticles (NPs) were synthesized in formic acid aqueous solutions through chemical reduction. Formic acid was used for a reducing agent of Ag precursor and solvent of gelatin. Silver acetate, silver tetrafluoroborate, silver nitrate, and silver phosphate were used as Ag precursors. Ag^+^ ions were reduced into Ag NPs by formic acid. The formation of Ag NPs was characterized by a UV-Vis spectrophotometer. Ag NPs were quickly generated within a few minutes in silver nitrate (AgNO_3_)/formic acid solution. As the water content of formic acid aqueous solution increased, more Ag NPs were generated, at a higher rate and with greater size. When gelatin was added to the AgNO_3_/formic acid solution, the Ag NPs were stabilized, resulting in smaller particles. Moreover, gelatin limits further aggregation of Ag NPs, which were effectively dispersed in solution. The amount of Ag NPs formed increased with increasing concentration of AgNO_3_ and aging time. Gelatin nanofibers containing Ag NPs were fabricated by electrospinning. The average diameters of gelatin nanofibers were 166.52 ± 32.72 nm, but these decreased with the addition of AgNO_3_. The average diameters of the Ag NPs in gelatin nanofibers ranged between 13 and 25 nm, which was confirmed by transmission electron microscopy (TEM).

## 1. Introduction

The application of nano-scale materials and structures, usually ranging from 1 to 100 nanometers (nm), is an emerging area of nanoscience and nanotechnology. Nanomaterials may provide solutions to technological and environmental challenges in the areas of solar energy conversion, catalysis, medicine, and water treatment [1,2]. Nanomaterials often show unique physical, chemical, and biological properties that are changed considerably compared to their macro-scaled counterparts [3]. One area of constant interest is the synthesis of noble-metal nanoparticles (NPs) for applications such as catalysis, electronics, optics, environmental applications, and biotechnology [4]. Gold, silver, and copper have been used most for the synthesis of stable dispersions of NPs, which are useful in areas such as photography, catalysis, biological labeling, photonics, optoelectronics, and surface-enhanced Raman scattering (SERS) detection [5,6].

Generally, metal NPs can be prepared and stabilized by various physical and chemical methods. Chemical approaches such as chemical reduction, electrochemical techniques, and photochemical reduction are most widely used [7,8]. Studies have shown that the size, morphology, stability, and properties (chemical and physical) of metal NPs are strongly influenced by the experimental conditions, the kinetics of interaction of metal ions with reducing agents, and the adsorption processes of the stabilizing agent with metal NPs [9,10]. Hence, the design of a synthesis method, in which these parameters can be controlled, has become a major field of interest [11].

Silver (Ag) is well known as a catalyst for the oxidation of methanol to formaldehyde and ethylene to ethylene oxide [12]. Ag is also of particular interest because of its distinctive properties, such as good conductivity, chemical stability, and catalytic and antibacterial activity [8]. Ag NPs have been prepared by various methods, such as chemical reduction, electrochemical reduction, templates, γ-ray or light irradiation reduction, biological synthesis, and microwave reduction.

Chemical reduction is the method most frequently applied for the preparation of Ag NPs as stable, colloidal dispersions in water or organic solvents. The reductants commonly used are borohydride, citrate, ascorbate, and elemental hydrogen. The reduction of Ag ions (Ag^+^) in aqueous solution generally yields colloidal Ag with particle diameters of several nanometers [13,14]. Initially, the reduction of various complexes with Ag^+^ ions leads to the formation of Ag atoms (Ag^0^), which is followed by agglomeration into oligomeric clusters. These clusters eventually lead to the formation of colloidal Ag particles [15]. When the colloidal particles are much smaller than the wavelength of visible light, the solutions are yellow in color with an absorption spectrum in a wavelength range of 380–400 nm. This band is attributed to the collective excitation of the electron gas in the particles, with a periodic change in electron density at the surface (surface plasmon absorption) [16–18]. Previous studies showed that the use of a strong reductant such as borohydride resulted in small particles that were somewhat monodisperse, but the generation of larger particles was difficult to control. The use of a weaker reductant such as citrate resulted in a slower reduction rate, but the size distribution was far from narrow [19–21].

The syntheses of NPs by chemical reduction methods are therefore often performed in the presence of stabilizers in order to prevent unwanted agglomeration of the NPs. Ag NPs in aqueous solutions are not stable, and rapidly undergo agglomeration due to the high reactivity of Ag NPs. However, this agglomeration can be reduced significantly if the stabilization of NPs is achieved using appropriate stabilizing agents. Electrostatic or steric stabilization techniques have been investigated to isolate discrete Ag NPs from solution. These include coating NPs using thiol compounds, encapsulation of the particles in micro-emulsions, polymer assemblies, or using dendritic structures. Capping or protective agents not only protect the NPs from precipitation, but also play a critical role in determining the size, size distribution, morphology, and the biocompatibility of the resulting Ag NPs [22].

A wide range of polymers has also been investigated as stabilizing agents for Ag NPs. Poly(vinyl pyrrolidone) (PVP) is one excellent polymeric stabilizing agent for Ag NPs. Other polymers including polyacrylates, poly(vinyl alcohol) (PVA), polyacrylonitrile, polyacrylamide, cellulose acetate, chitosan and alginate have been investigated as stabilizing molecules for Ag NPs [23–26]. Patakfalvi *et al.* [27] evaluated the effect of different polymers and polymer concentration on the reaction rate and size distribution of Ag NPs prepared from aqueous AgNO_3_ solution, using hydroquinone and sodium citrate as reducing agents. The stabilizing agents used were PVP and PVA. The study showed that PVP polymer chains provided more effective steric stabilization and reduced the growth rate of Ag NPs compared to PVA. Also, the size of the Ag NPs was significantly influenced by the polymer concentration. Higher polymer concentrations led to smaller particle size. In addition to the size of the particles, the concentration of PVP significantly affected the shape of the Ag NPs.

Gelatin is a naturally abundant biopolymer, and has been known to have biocompatibility and biodegradability similar to collagen. Gelatin can be easily obtained by extraction from animal tissue such as skin, muscle, and bone. Due to its natural abundance and inherent biodegradability in physiological environments, gelatin is widely used in food, cosmetic, pharmaceutical, and medical applications. Depending on its application, gelatin can be fabricated in many forms, including films, micro- or nanoparticles, and dense or porous hydrogels. Recently, gelatin-based scaffolds prepared by electrospinning have been investigated intensely, because their three-dimensional (3D) structure mimics extracellular matrix very well, which makes them very attractive for tissue engineering applications [28–35].

Electrospinning has been studied widely because of its efficiency and simplicity for the fabrication of nanofibrous structures. The nanofibers exhibit outstanding characteristics, such as very large surface-area-to-volume ratio and high porosity with very small pore size. Electrospun nanofibers are candidate materials for many biomedical applications, such as wound dressings, drug delivery, and scaffolds for tissue engineering [36–41].

Generally, gelatin can be dissolved in warm water and fabricated in various forms, but it has not been possible to obtain gelatin nanofibers by electrospinning, because aqueous gelatin solutions easily turn to gel in the syringe needle at room temperature. Because of these limitations associated with using water as a solvent, the electrospinning of gelatin requires the use of quickly evaporating organic solvents. 2,2,2-trifluoroethanol (TFE), 1,1,1,3,3,3-hexafluoro-2-propanol (HFIP), formic acid, and acetic acid are suitable solvents for preparing an electrospinnable gelatin solution [42–49]. Because of the inherent properties of gelatin and the unique characteristics of the electrospun fibers, electrospun gelatin fibers are ideal materials for use as scaffolds for cell and tissue culture, carriers for topical/trans dermal delivery of drugs, and wound dressings [28,44,46,47,50].

Ag NPs can be incorporated into polymer matrices, including polymer nanofibers. The incorporation of Ag NPs into a nanofibrous structure is an attractive method, because Ag NPs distributed evenly in a nanofibrous structure can be used in novel specific applications in catalysis, sensors, photonics, and electronics [51–55]. Many studies have examined Ag salts or Ag compounds as promising materials for wound management. Ag salts or Ag compounds have been used for their antimicrobial activity in wound treatments in a variety of physical forms, such as beads, gels, films, and fibers. Recently, there has been a rapid increase in the number of commercial Ag dressings available, such as silver nitrate, silver sulphadiazine, and nanocrystalline silver [56–60].

**Figure 1.**
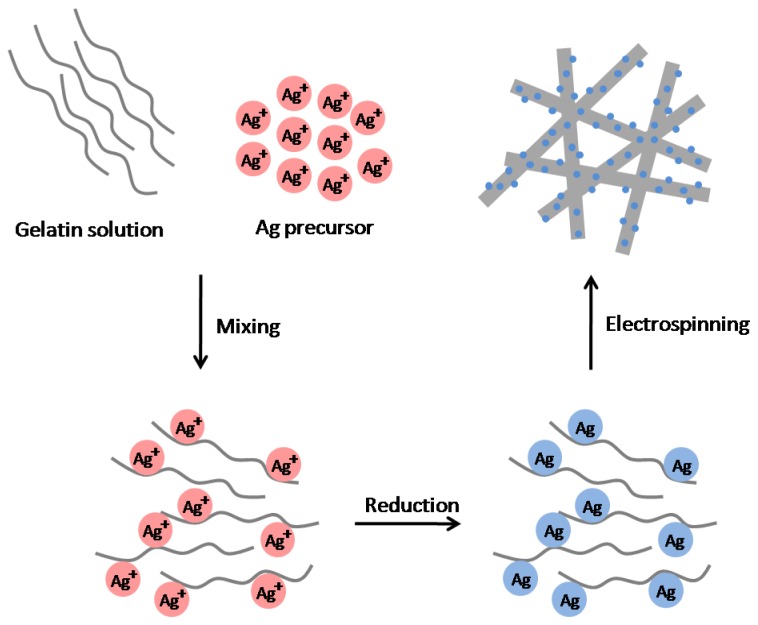
Schematic of the one-step process for fabricating Ag NPs-polymer nanofiber composites via electrospinning.

A combination of Ag NPs and polymer nanofibers can be obtained by electrospinning polymer solutions containing Ag NPs or Ag salts, which are then reduced into particles in electrospun polymer nanofibers. Figure 1 is a schematic representing the procedure for the one-step synthesis of Ag NPs-polymer nanofiber composites coupled with electrospinning [61]. A number of methods have been used to generate Ag NPs from Ag ions in electrospun fibers, including chemical reduction by hydrazinium hydroxide or DMF; photocatalytic reduction by TiO_2_ NPs; photoreduction by UV irradiation; and simple heat treatment. In these methods, the as-formed Ag NPs are prevented from further growth and agglomeration (*i.e.*, stabilized) by polymer matrix [62–69].

In this study, Ag NPs were synthesized through chemical reduction. Silver acetate (CH_3_COOAg), silver tetrafluoroborate (AgBF_4_), silver nitrate (AgNO_3_), and silver phosphate (Ag_3_PO_4_) were used as Ag precursors, and dissolved into formic acid/water solutions. The synthesis of Ag NPs was characterized by UV-Vis spectrophotometry. Gelatin was used as a stabilizer, and the size distribution and dispersion of Ag NPs formed in gelatin solution were characterized using a UV-Vis spectrophotometer, nanoparticle analyzer, and transmission electron microscopy (TEM). Also, gelatin nanofibers containing Ag NPs were prepared by electrospinning to explore the nanofibrous scaffolds for burn wound dressing. Their morphology was investigated by field emission scanning electron microscopy (FE-SEM). Ag NPs in gelatin nanofibers were observed using a transmission electron microscope (TEM) and confirmed by energy dispersive spectroscopy (EDS).

## 2. Results and Discussion

### 2.1. The Formation of Ag NPs in Formic Acid/Water Solution

In the chemical reduction method, Ag precursor was dissociated from Ag^+^ ions in aqueous solution, and Ag^+^ ions were reduced to Ag^0^ (Ag NPs) by a reducing agent. In this system, various Ag compounds were used as an Ag precursor, such as CH_3_COOAg, AgBF_4_, AgNO_3_, and Ag_3_PO_4_. Formic acid was used as a solvent and reducing agent of Ag precursor at the same time. Ag NPs have a surface plasmon resonance (SPR) absorption in the UV-visible region. The SPR band arises from the coherent existence of free electrons in the conduction band due to the small particle size. The band shift is dependent on the particle size, chemical surrounding, adsorbed species on the surface, and dielectric constant. A unique characteristic of these synthesized Ag particles is that a change in the absorbance or wavelength gives a measure of the particle size, shape, and interparticle properties [4].

**Figure 2.**
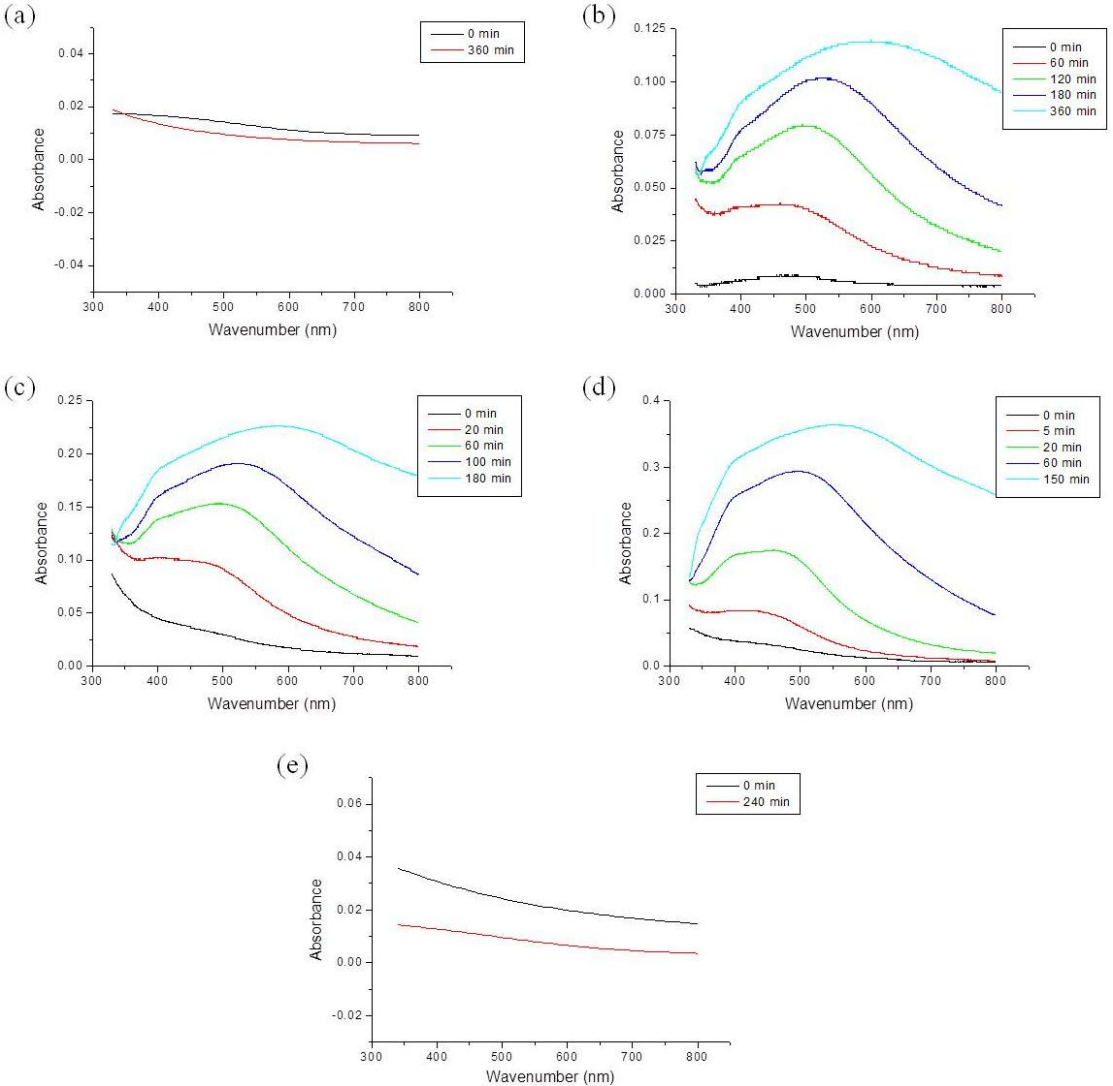
UV-Vis absorption spectra of silver acetate in acidic solution: (**a**) F100; (**b**) F70W30; (**c**) F50W50; (**d**) F30W70; (**e**) W100.

**Figure 3.**
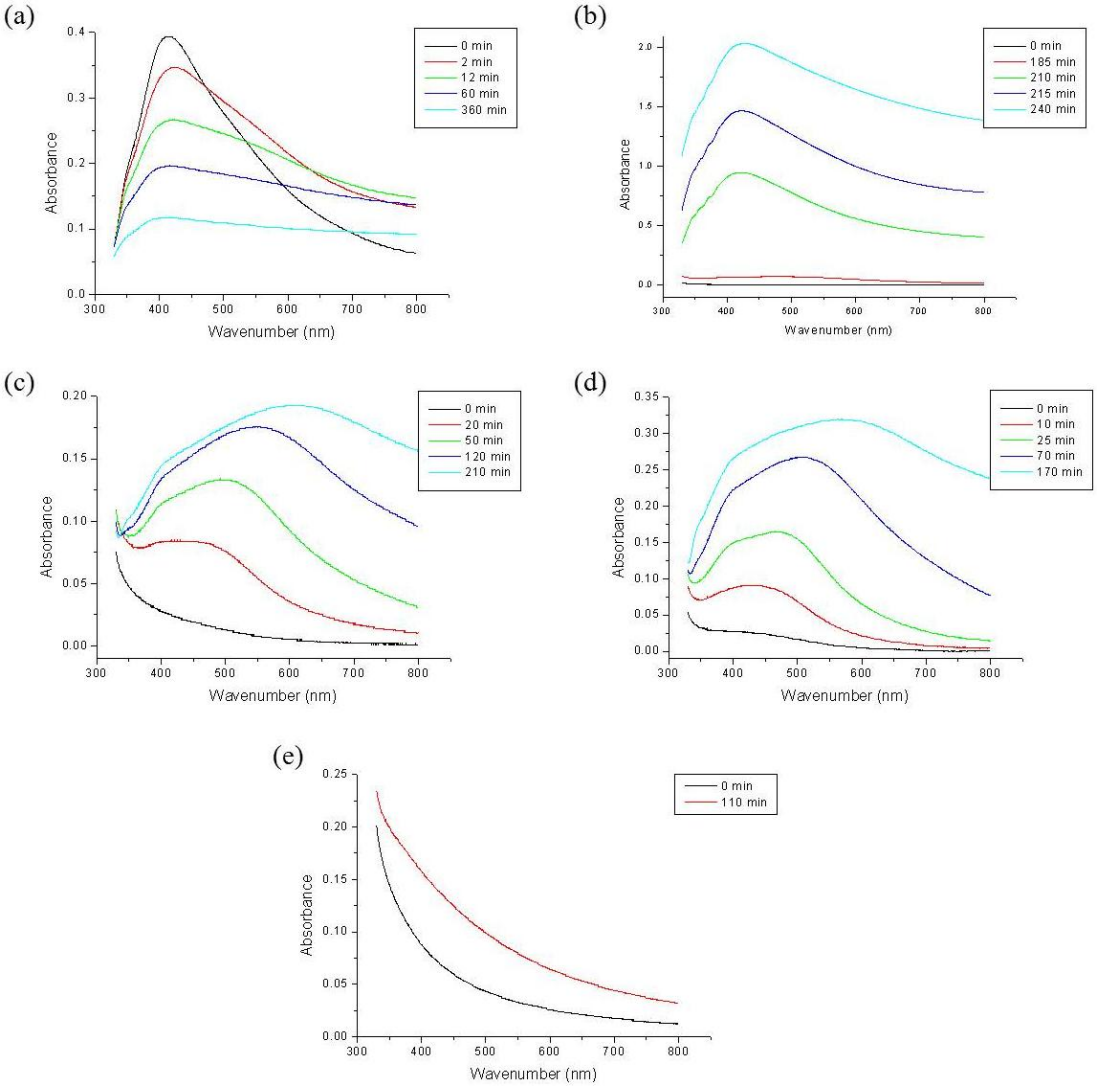
UV-Vis absorption spectra of silver nitrate in acidic solution: (**a**) F100; (**b**) F70W30; (**c**) F50W50; (**d**) F30W70; (**e**) W100.

The UV-Vis spectra show the formation and growth of Ag NPs in acidic solution, as shown in Figures 2 and 3 and Figures S1 and S2. Figure 2 shows the UV-Vis absorption spectra of CH_3_COOAg in acidic solution. CH_3_COOAg was easily dissolved in all compositions of formic acidic solutions: F100 (formic acid 100%), F70W30 (formic acid 70%/H_2_O 30%), F50/W50 (formic acid 50%/H_2_O 50%), F30W70 (formic acid 30%/H_2_O 70%), and W100 (H_2_O 100%). In F70W30 solution, the absorption band was observed in the region of 400–600 nm, and the absorbance was increased with reaction time. As the concentration of formic acid decreased, the absorbance was increased, and the peak positions of the maximum absorption peak, λ_max_, were shifted to a longer wavelength. λ_max_ was dependent on the growth rate of the Ag NPs, and the value of *d*λ_max_*/dt* reflected the reaction rate. The shift in the absorption band to a longer wavelength indicates that the particle size was increased. However, CH_3_COOAg was not reduced in F100 and W100.

The UV-Vis absorption spectra of AgBF_4_ in acidic solution were observed to be similar to the spectra of CH_3_COOAg (Figure S1). AgBF_4_ was also easily dissolved in all formic acidic solutions. In the F70W30 solution, the absorption band was observed in the same region, and the absorbance also increased with the reaction time. As the concentration of formic acid decreased, the absorbance was increased, λ_max_ shifted to a longer wavelength, and the time to λ_max_ was shorter. However, AgBF_4_ was also not reduced in F100 and W100.

Figure 3 shows the UV-Vis absorption spectra of NaNO_3_ in acidic solution. Unlike the spectra of CH_3_COOAg or AgBF_4_, the strongest absorption band was observed at 420 nm for the F100 solution. With the F70W30 solution, the absorption band was observed in the same region, but the absorbance was higher than that of CH_3_COOAg or AgBF_4_ in the same solution. This means that the Ag^+^ ions of AgNO_3_ were rapidly reduced to Ag NPs, and formed many Ag NPs in F100 and F70W30 solutions. However, the absorbance of the peak was decreased with the reaction time, indicating that the formed Ag NPs were precipitated. Therefore, the syntheses of NPs by chemical reduction methods are often performed in the presence of stabilizers in order to prevent unwanted precipitation and agglomeration of the NPs.

The UV-Vis absorption spectra of Ag_3_PO_4_ in mixed acidic solutions were observed to be similar to those of CH_3_COOAg and AgBF_4_ (Figure S2). Because the Ag_3_PO_4_ was not dissolved in water (W100), the UV-Vis absorption spectra were not available.

**Figure 4.**
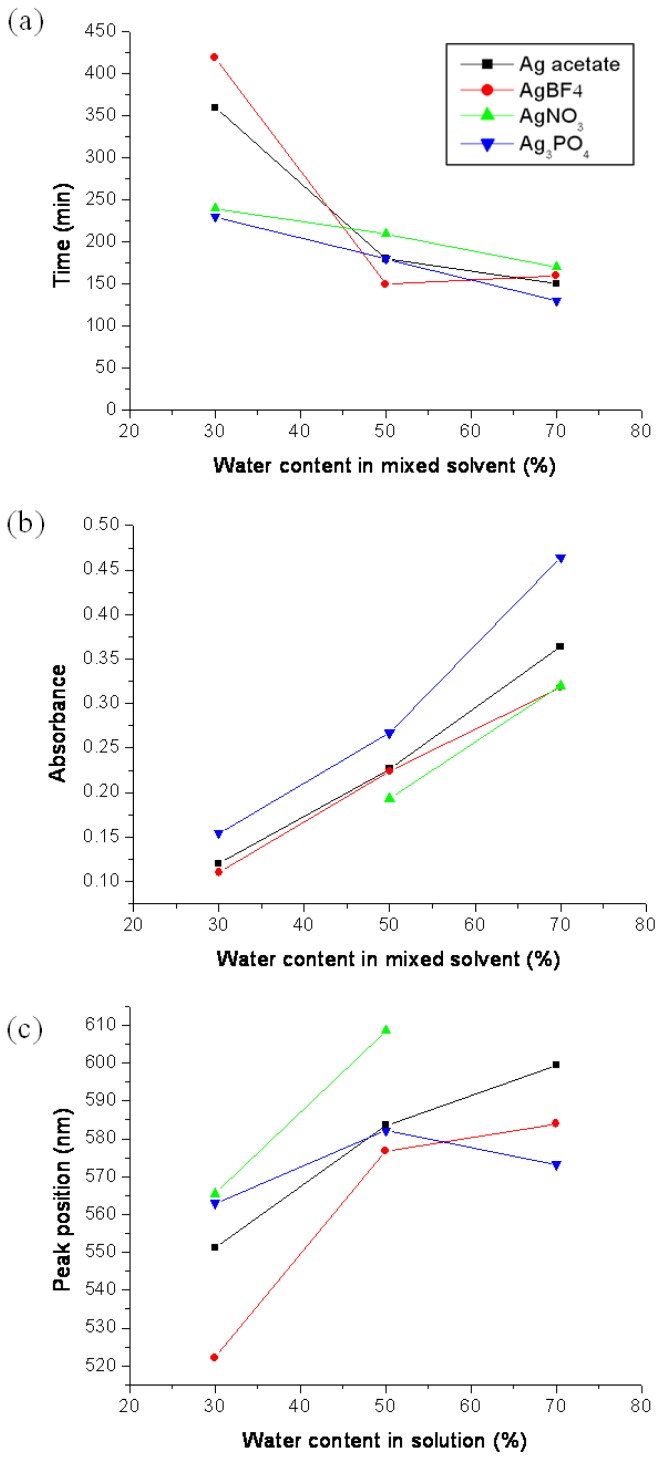
Relationship of (**a**) the time to maximum absorption peak; (**b**) the maximum absorbance; (**c**) peak position of maximum absorption peak *vs*. water content.

Overall, when Ag precursor was dissolved in formic acidic solution, Ag^+^ ions that dissociated from the Ag precursor were reduced to Ag NPs by formic acid. The strongest absorption band was observed in the UV-Vis spectra of AgNO_3_ in F100 solutions, indicating that a number of Ag NPs were formed in the solution. This could be explained by the higher reducing ability of AgNO_3_. The relationship between the maximum absorbance, peak position of the maximum absorption, time to the maximum absorption peak, and water content are summarized in Figure 4.

### 2.2. Role of Formic Acid in the Formation of Ag NPs

Formic acid is dissociated in aqueous solution as follows (Equation (1)):

(1) $$\text{HCOOH }(\text{aq})↔{\text{H}}^{+}+{\text{HCOO}}^{-}$$

The formic acidic solutions of various compositions were prepared and its conductivity was analyzed using a conductivity meter. The conductivity was increased with increasing water content (Figure S3). The higher conductivity indicates that the solution contained a high amount of dissolved ions. Thus, it could be thought that higher dissociated formate anions (HCOO^−^) existed in the solution with higher-conductivity.

The Ag precursor was dissociated from Ag^+^ ions in aqueous solution. Ag^+^ ions may lead to the formation of Ag-formate complex, which can be expressed by Equations (2) and (3):

(2) $${\text{Ag}}^{+}\;{\text{HCOO}}^{-}↔{\text{Ag}}^{+}+{\text{HCOO}}^{-}$$

in formic acidic solution,

(3) $${\text{Ag}}^{+}+{\text{HCOO}}^{-}↔[\text{Ag}\ldots \text{OOCH}]$$

Ag-formate complex grows by aggregation, which forms the Ag NPs.

Therefore, many Ag^+^ ions were more bonded with formate ions because there are many formate ions in F30W70 solutions, and then Ag NPs grow and aggregate further.

### 2.3. UV-Vis Analysis of Gelatin Solution Containing AgNO_3_

**Figure 5.**
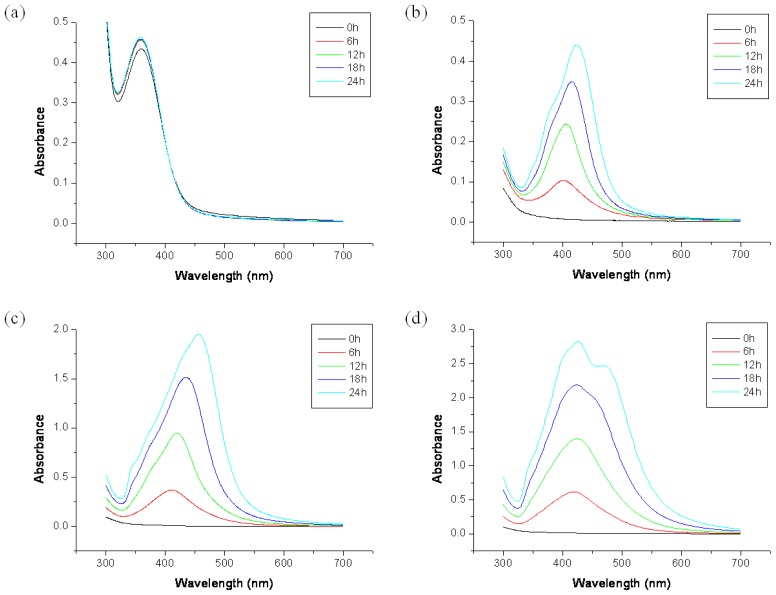
UV-Vis absorption spectra of gelatin solution containing AgNO_3_: (**a**) F100; (**b**) F70W30; (**c**) F50W50; (**d**) F30W70.

The time-resolved changes of UV-Vis absorption bands of gelatin/AgNO_3_ solutions with various solvent compositions were first investigated. As shown in Figure 5, the absorption band of Ag NPs in F100 gelatin solution was observed in the 360 nm region, and did not changed for 24 h. This means that the Ag NPs in the F100 solution in the presence of gelatin were very stable without growth or aggregation of the Ag NPs. This behavior differed substantially from that of Ag NPs in F100 solution (Figure 3), which precipitation easily occurred within a few minutes. However, in F70W30 gelatin solution, the UV-Vis absorption spectra gradually increased over time, and the peak position was shifted to a longer wavelength. The UV-Vis spectra of F50W50 gelatin solution were observed to be similar to that of F70W30, but its absorbance was higher than that of F70W30, and its absorption band was slightly shifted to a longer wavelength. This means that in the F50W50 solution, more Ag NPs with greater size were generated than in the F70W30 solution. In the F30W70 gelatin solution, two absorption peaks were observed at 425 and 470 nm, which correspond to two particle populations of Ag NPs [70].

### 2.4. Particle Size Analysis of Ag NPs in Gelatin Solution

**Figure 6.**
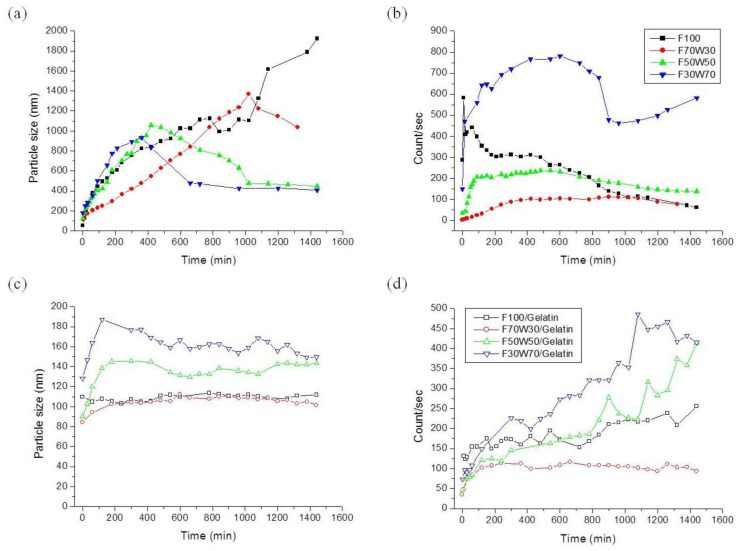
Comparison of (**a**) average particle size of Ag NPs and (**b**) detected number of Ag NPs in acidic solution without gelatin; Comparison of (**c**) average particle size of Ag NPs and (**d**) detected number of Ag NPs in gelatin solution.

The average particle size of Ag NPs was characterized using a nanoparticle size analyzer. The nanoparticle size analyzer is an innovative photon cross-correlation sensor that allows for the simultaneous measurement of particle size and the stability of opaque emulsions and suspensions in the nanometer region. Figure 6 shows the time-resolved changes in particle size and counts in gelatin/AgNO_3_ solutions with various solvent compositions. The size of Ag NPs in the F100 solution was gradually increased to 2 μm, but the detected particle counts decreased over time. The average particle size in F70W30 solution was also increased to 1.4 μm, and the counted particles decreased compared to the F100. The average particle size in the F50W50 and F30W70 solutions increased to a maximum of about 1 μm, and then decreased to 400 nm. The Ag NPs synthesized in formic acidic solution were easily aggregated, because there was no stabilizer to prevent aggregation. If the Ag NPs experienced greater growth and aggregation, the aggregates became heavy and were precipitated. This made the deposited aggregates too difficult to detect with a nanoparticle size analyzer, so the average particle diameter decreased.

When gelatin was added in aqueous solution, the average particle size was decreased significantly to about 200 nm. In gelatin/F100 and gelatin/F70W30 solutions, the average particle diameter was not changed for 24 h. This indicates that the Ag NPs were capped with gelatin, which prohibited the aggregation of Ag NPs. Therefore, the effect of gelatin as a stabilizer was confirmed by nanoparticles analysis, which matched with the results of UV-Vis spectra. The particle size and count was affected by the composition of the acidic solution, as shown in Figure 6. As mentioned earlier, in the F30W70 solution, many Ag NPs were generated. A relationship between formate ions and Ag NPs can be induced from the nanoparticle analysis results. As shown in Figure 6, there were more particles detected in F30W70 than in other solution compositions, even in aqueous gelatin solutions.

**Figure 7.**
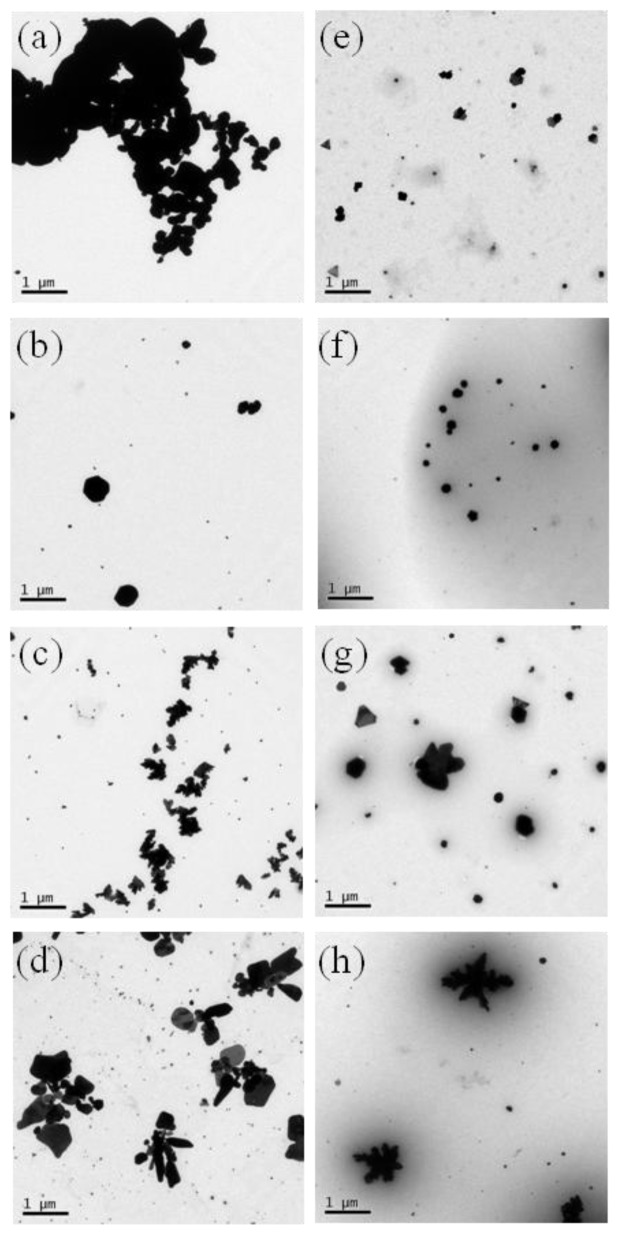
TEM images of Ag NPs in acidic solution: (**a**) F100; (**b**) F70W30; (**c**) F50W50; (**d**) gelatin/F30W70; (**e**) gelatin/F100; (**f**) gelatin/F70W30; (**g**) gelatin/F50W50; (**h**) gelatin/F30W70.

The morphology of the Ag NPs in gelatin solution was confirmed using TEM. TEM images in Figure 7 show the Ag NPs synthesized in acidic solution. The shape and size of the synthesized Ag NPs varied depending on the addition of gelatin and the formic acid concentration (Figure S4). The Ag NPs in the F100 solution were substantially aggregated (Figure 7a), but Ag NPs in the gelatin/F100 solution were separately stabilized, with an average diameter of 103 ± 65 nm (Figure 7e). As the water content increased, the Ag NPs became larger and aggregated. These phenomena were observed in gelatin-stabilized Ag NPs, and were related to the formate ions and the viscosity of the gelatin solutions.

**Figure 8.**
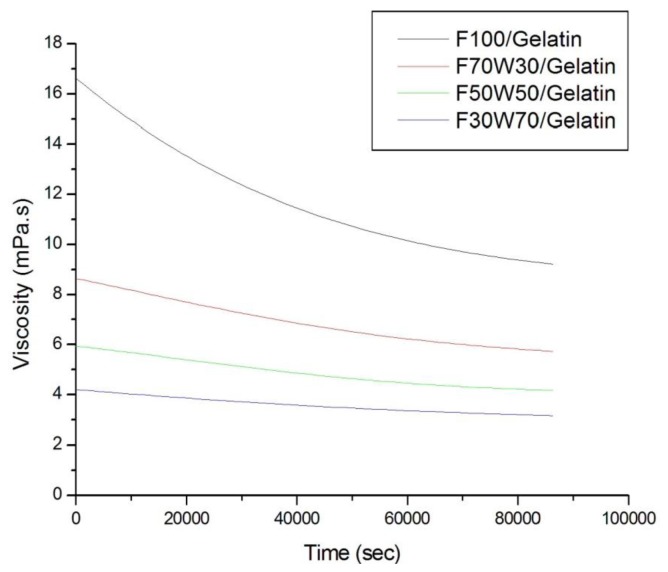
Viscosity of gelatin/formic acidic soluitons *vs*. composition of formic acid solutions.

The viscosity of gelatin might be affected by the composition of the aqueous solution and the gelatin concentration. Shin *et al.* [71] investigated the growth mechanism of colloidal Ag NPs stabilized by PVP. In their results, the average size of the Ag NPs decreased with the increasing amounts of PVP. If the viscosity of the polymer solution was decreased, the Ag NPs could easily come into contact with other Ag molecules. Further aggregation occurred, and the size of Ag NPs increased. As shown in Figure 8, the viscosity of the gelatin solution was decreased depending on the time, the composition of formic acid and water, and the gelatin concentration. Since the viscosities of the F50W50 and F30W70 gelatin solutions were lower than that of F70W30, the average particle size was increased, and the Ag NPs were aggregated.

### 2.5. Formation of Ag NPs in Gelatin Solution

UV-Vis absorption spectra are known to be quite sensitive to the formation of Ag NPs. Light with an appropriate wavelength can cause oscillation in the conduction electrons of Ag NPs. The interaction of light with the electrons of Ag NPs leads to a phenomenon known as SPR, which results in optical SPR absorption peaks [72]. Gelatin solutions containing Ag NPs show characteristic optical absorption spectra in the UV-Vis region.

**Figure 9.**
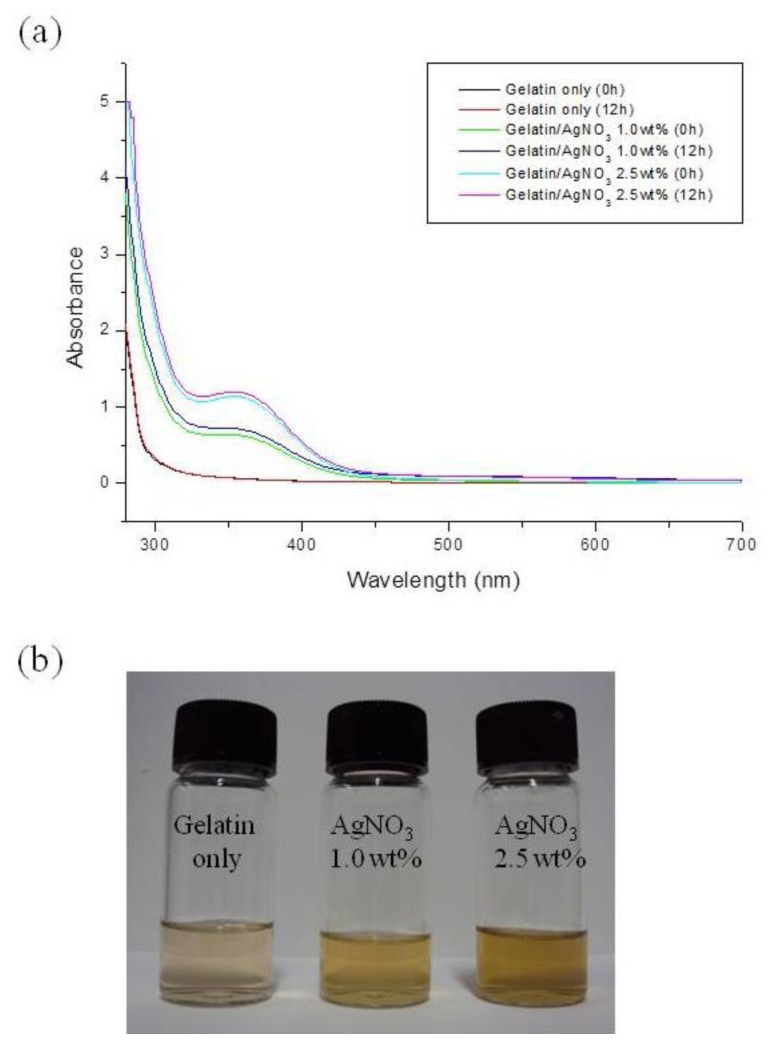
(**a**) UV-Vis absorption spectra of gelatin solution containing Ag NPs; (**b**) Appearance of gelatin solutions with different AgNO_3_ concentration.

The UV-Vis absorption spectra of gelatin solution containing Ag NPs are shown in Figure 9a. It can be seen that there was an absorption band with a peak around 320–450 nm, which corresponded to the SPR absorption band of the Ag. An increase in the AgNO_3_ concentration resulted in an observed increase in the intensity of the bands. Additionally, the absorbance of 12-h-aged gelatin solution containing AgNO_3_ was slightly increased. This indicated that the number of Ag^+^ ions that were converted into Ag NPs increased with an increase in the AgNO_3_ concentration and aging time. No absorption of any kind was observed for the base gelatin solution.

The reduction of Ag^+^ ions into elemental Ag (*i.e.*, Ag NPs) in the AgNO_3_ containing gelatin solutions at different AgNO_3_ concentration could be visualized from changes in the color of the solutions, from light yellow to yellow, as shown in Figure 9b. This color change is associated with an increase in the absorbance with increasing Ag concentration. The as-formed Ag NPs were prevented from further growth and agglomeration by the gelatin. Ag^+^ ions were reduced directly into Ag NPs through a series of steps, including nuclei formation, crystal growth via diffusion mechanism to give primary particles, and spontaneous self-organization of primary particles to form clusters. The formation of the Ag NPs nuclei was postulated to originate from the ionic interactions between Ag^+^ ions and either –NH_2_ or –COOH groups, or both, on gelatin chains, according the following schemes (Equations (4) and (5)):

(4) H2N-COOH+AgNO3↔H2N-COO Ag+HNO3

(5) H2N-COOH+AgNO3↔AgHN-COOH+HNO3

or from simple binding between Ag^+^ ions and lone pair electrons of N and O atoms in the –NH_2_ and –COOH groups, followed by the reduction of Ag^+^ ions into Ag NPs nuclei [73].

### 2.6. Electrospinning of Gelatin Solution Containing Ag NPs

Gelatin is very easily dissolved in warm water, but aqueous gelatin solutions change to a gel at room temperature. Furthermore, water cannot be volatilized quickly enough to coagulate the gelatin in electrospinning processes. Formic acid, a volatile organic solvent, is a suitable for overcoming these weak points. A high amount of gelatin can be dissolved in formic acid at room temperature [74].

The viscosity of a polymer solution usually depends on the concentration of the polymer. The viscosity of gelatin dissolved in formic acid increased with increasing of gelatin concentration. The viscosity of gelatin increased rapidly at ~10 wt %, indicating that extensive chain entanglement of the gelatin occurred around this concentration. This means that the liquid can be ejected, and fibers would form as the solvent evaporates.

Gelatin solution containing AgNO_3_ was loaded into a 10-mL plastic syringe, which was attached to a blunt 24-gauge stainless steel needle. The syringe with the solution loaded was placed in a syringe pump. Electrospinning was performed with various concentrations ranging from 6–16 wt % under an electric field of 2.0 kV/cm, with a tip-to-collector distance of 10 cm. The mass flow rate of solutions was 0.4 mL/h. At a lower concentration (below 8 wt %), the electrospun nanofibers could not be formed, and large beads were generated. As the gelatin concentration increased, the electrospun nanofibers were changed to a continuous fibrous structure and randomly arranged with nanometer-scale diameter at concentrations of 10–16 wt %. The average diameter of electrospun gelatin nanofibers gradually increased with increasing of gelatin concentration. From an image analysis, the electrospun fiber diameters of the 16% gelatin solution were measured with an average of 166.52 nm.

**Figure 10.**
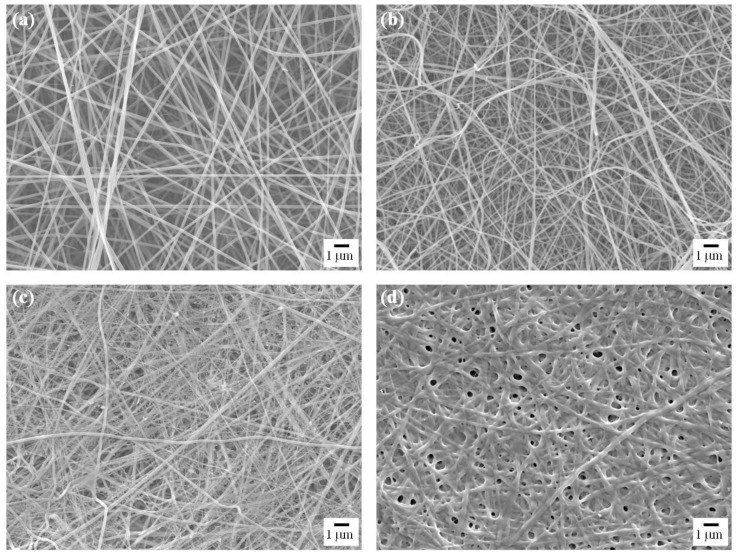
SEM images of as-spun gelatin nanofibers containing Ag NPs prepared with different contents of AgNO_3_: (**a**) 0 wt %; (**b**) 1.0 wt %; (**c**) 2.5 wt %; (**d**) 24 h cross-linked gelatin nanofibers containing Ag NPs. Scale bar indicates 1 μm.

Figure 10 shows SEM images of gelatin nanofibers electrospun from 16 wt % gelatin with different contents of AgNO_3_. The average fiber diameters of nanofibers containing Ag NPs decreased than gelatin nanofibers. The average fiber diameters of 166.52 ± 32.72, 117.51 ± 28.39 and 115.38 ± 49.88 nm corresponding to 0, 1.0, and 2.5 wt % AgNO_3_ content were measured, respectively. Generally, the viscosity and surface tension of a polymer solution are not significantly changed with addition of salts, although it’s the conductivity is affected considerably. The conductivity of the pure gelatin solution was 2.24 mS/cm, and it increased to 2.64 and 2.89 mS/cm after the addition of 1.0 and 2.5 wt % AgNO_3_ into the solution, respectively. In the results, stronger elongation forces imposed on the ejected jets under an electrical field corresponded to straighter and finer fibers produced [74].

### 2.7. Cross-Linking of Gelatin Nanofibers Containing Ag NPs

Since gelatin is water-soluble, the electrospun gelatin fibers can easily dissolve either partially or completely, losing their fibrous structure when coming into contact with an aqueous medium. The fibers may partially dissolve and lose their fibrous structure upon exposure to high ambient humidity (e.g., 80%–90%) for a certain period of time [75]. To extend the use of electrospun gelatin fibers in applications that require exposure to an aqueous medium or high humidity, further cross-linking is necessary. Among the various chemical systems used to cross-link electrospun gelatin fibers (e.g., 1,6-diisocyanatohexane (HDMI), 1-ethyl-3-[3-dimethylaminopropyl] carbodiimide hydrochloride (EDC), and glutaraldehyde), glutaraldehyde seems to be the most suitable, as it is economical and does not compromise the fibrous structure [46,47,49,75]. Here, the electrospun gelatin fibers were cross-linked by saturated vapor from 50% glutaraldehyde aqueous solution for 48 h.

Figure 10d shows SEM images of cross-linked gelatin nanofibers containing Ag NPs. Exposing the fiber mats in the chamber caused some fibers to fuse to one another at contacting points, which is a result of the partial dissolution of the fiber segments when they came into contact with the moisture-rich glutaraldehyde vapor [27]. After cross-linking, the color of the electrospun gelatin nanofibers was changed from white to yellow (gelatin only) or brown (containing Ag NPs). The fibers also shrunk slightly from their original dimensions. The change in color of the gelatin upon cross-linking with glutaraldehyde is caused by the formation of aldimine linkages (–CH=N–) between the free amino groups of lysine or hydroxyl lysine amino acid residues of the protein and the aldehyde groups of glutaraldehyde [76,77]. The shrinkage of the fiber mats is responsible for the observed decrease in the size of inter-fibrous pores.

### 2.8. Characterization of Ag NPs in Gelatin Nanofibers

**Figure 11.**
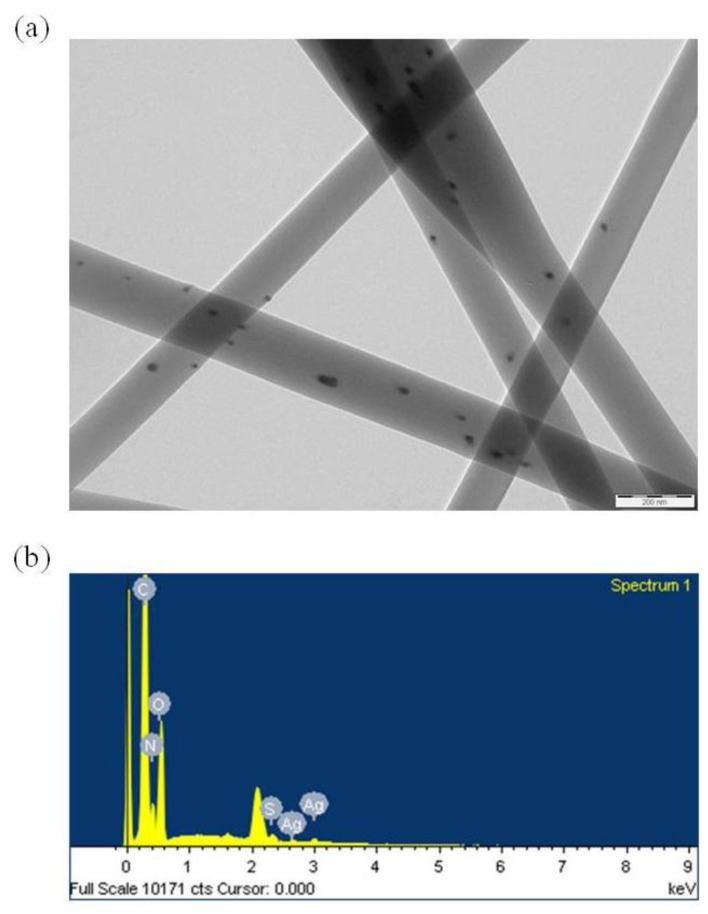
(**a**) TEM images and (**b**) energy dispersive spectroscopy (EDS) pattern of gelatin nanofibers containing Ag NPs. Scale bar indicates 200 nm.

Figure 11a shows a TEM image of gelatin nanofibers containing 1.0 wt % AgNO_3_. The Ag NPs were distributed throughout the fibers, with diameters of 19.6 ± 3.43 nm. The Ag NPs were well stabilized by the gelatin fibers during the electrospinning process. In EDS analysis, the Ag element was detected in the gelatin nanofibers containing Ag NPs, as shown in Figure 11b.

## 3. Experimental Section

### 3.1. Materials

Four types of Ag compounds were used: silver acetate (C_2_H_3_O_2_Ag; 99%), silver tetrafluoroborate (AgBF_4_; 98%), silver nitrate (AgNO_3_; >99.9%), and silver phosphate (Ag_3_PO_4_; 98%). Silver acetate, silver tetrafluoroborate, and silver phosphate were purchased from Sigma-Aldrich (St. Louis, MO, USA). Silver nitrate was purchased from Kojima Chemicals (Saitama, Japan). Formic acid (98%) was purchased from Junsei (Tokyo, Japan). Gelatin powder (type A from porcine skin) was purchased from Sigma-Aldrich (St. Louis, MO, USA). An aqueous solution of glutaraldehyde (50%) was purchased from Sigma-Aldrich (St. Louis, MO, USA). Glycine was purchased from Sigma-Aldrich (St. Louis, MO, USA). All chemicals were used without further purification.

### 3.2. Synthesis of Ag NPs

Ag NPs in acidic solution were prepared by the chemical reduction of Ag precursor. First, acidic solutions of various compositions were prepared: 98% formic acid only (F100), formic acid 70%:water 30% (*v*/*v*) (F70W30), formic acid 50%:water 50% (*v*/*v*) (F50W50), formic acid 30%:water 70% (*v*/*v*) (F30W70), distilled water only (W100). The 4 types of Ag compound were dissolved in formic acid/water solutions, each with a concentration of 0.3 wt %. The mixture was rapidly stirred with a vortex machine for 1 min at room temperature. In this system, formic acid was used as both a solvent for the Ag precursor and as a reducing agent for the Ag ions.

Ag NPs in gelatin solution were prepared by the chemical reduction of AgNO_3_. Gelatin was dissolved in a solution of formic acid/water with a concentration of 2 wt %. The gelatin/formic acid/water solution was stirred for 2 h at room temperature. Then, AgNO_3_ powder was added as an Ag precursor and dissolved into the gelatin/formic acid/water solution at a concentration of 0.3 wt %. The mixture was vortexed for 1 min at room temperature to allow the diffusion of Ag ions into the gelatin/formic acid solution. In this system, gelatin was used as a stabilizer and dispersing agent for the Ag NPs.

### 3.3. Preparation of Gelatin Nanofibers Containing Ag NPs

In the electrospinning process, the solvent in the polymer solution has a predominant influence on its spinnability. Although gelatin can be dissolved in water at temperatures over 40 °C, the aqueous gelatin solution cannot be electrospun into ultra-fine fibers at room temperature [45,74]. Formic acid was chosen as a co-solvent for gelatin and AgNO_3_ in this study. Gelatin was first dissolved in 98% formic acid with concentrations of 6–16 wt %, and AgNO_3_ was then added to the gelatin/formic acid solution. The concentration of AgNO_3_ was 0.1–2.5 wt % of the gelatin weight. The gelatin solution containing AgNO_3_ was loaded into a 10-mL plastic syringe, which was attached to a blunt 24-gauge stainless steel needle. The syringe with the solution loaded was placed in a syringe pump (model 220, KD Scientific, Hollistone, MA, USA). A 20-kV voltage was applied by a high-voltage power supply to the collecting target, which was wrapped around a rotating cylinder with aluminum foil (CPS-60K02VIT, Chungpa EMT, Seoul, Korea). The mass flow rate of solutions was 0.4 mL/h. The distance between the needle tip and the collecting target was kept at 10 cm. All the electrospinning procedures were carried out at room temperature. In order to improve the water-resistance of gelatin nanofibers, the electrospun gelatin nanofibers containing Ag NPs were cross-linked by treatment with glutaraldehyde vapor, saturated with 50% glutaraldehyde aqueous solution, at room temperature for various durations. After cross-linking, gelatin nanofibers containing Ag NPs were treated with 0.1 M glycine aqueous solution to block unreacted aldehyde groups, and dried in a freeze-dryer.

### 3.4. Characterization

The existence and formation behavior of the Ag NPs in the formic acid/water mixed solutions were confirmed by the surface plasmon absorption band using a UV-Vis spectrophotometer (UV-2450, Shimadzu, Kyoto, Japan), with a 2-mm rectangular quartz cell (5061–3385, Agilent Technologies, Waldbronn, Germany). The size distribution and dispersion of Ag NPs formed in gelatin solution were characterized by a nanoparticle analyzer (NANOPHOX, Sympatec GmbH, Clausthal-Zellerfeld, Germany). The morphology of the Ag NPs formed in gelatin solution was confirmed using a transmission electron microscope (TEM) (Technai G2 Spilit, FEI Company, Hillsboro, OR, USA). The electrical conductivity of the mixed solvent was measured by a conductivity meter (455C, Istek, Seoul, Korea). The viscosity of gelatin solution was measured at room temperature using a vibro viscometer (SV-10, A&D Company, Tokyo, Japan).

The morphology of the electrospun gelatin nanofibers containing Ag NPs was observed using a field emission scanning electron microscope (FE-SEM) (JSM-7000F, JEOL, Tokyo, Japan) after platinum coating. The Ag NPs in gelatin nanofibers were confirmed through energy dispersive spectroscopy (EDS) (JSM-7000F, JEOL, Tokyo, Japan) and energy filtering transmission electron microscope (EF-TEM) (EM912 Omega, Carl Zeiss, Jena, Germany). The average diameter and diameter distribution of gelatin nanofibers and Ag NPs were obtained by analyzing FE-SEM images using an image analysis program (Scope Eye II, Visual Inspection Technology, Seoul, Korea).

## 4. Conclusions

Ag NPs were prepared in formic acidic solutions with various compositions, using silver acetate, silver tetrafluoroborate, silver nitrate, and silver phosphate as silver precursors. The Ag^+^ ions were reduced to Ag NPs by formic acid. In the silver nitrate/F100 solution, the Ag NPs were immediately generated within a few minutes, and the size and amount of the NPs increased gradually with time. UV-Vis spectra analysis confirmed the role of formic acid as follows: (1) Formic acid was dissociated from formate ions, which bonded with Ag^+^ ions; (2) As the water content increased, more Ag NPs were generated, at a faster rate and with larger size. If there are no materials to prevent aggregation, Ag NPs grow and further aggregate. However, when gelatin was added to the AgNO_3_/formic acid solution, the Ag NPs were stabilized, which resulted in smaller particles. Moreover, the gelatin limited the further aggregation of Ag NPs, and was effectively dispersed in the solution. Therefore, the syntheses of NPs by chemical reduction methods have to perform in the presence of stabilizers in order to prevent unwanted deposition and agglomeration of the NPs.

The gelatin nanofibers containing Ag NPs were prepared by electrospinning. The average diameter of the gelatin nanofibers was 166.52 ± 32.72 nm, which decreased with AgNO_3_. The average diameters of the Ag NPs in gelatin nanofibers ranged between 13 and 25 nm, which was confirmed by TEM. In EDS analysis, the Ag element was also detected in the gelatin nanofibers containing Ag NPs. The gelatin nanofibers containing Ag NPs were cross-linked, in order to improve their stability in an aqueous medium or a high-humidity atmosphere, by saturated vapor from a 50 vol % glutaraldehyde aqueous solution. Cross-linking caused the color of the gelatin nanofibers to change, and caused fusing and shrinking of the gelatin nanofibers.

## Supplementary Information
