# Supplementary material for: Preparation and Characterization of Gelatin Nanofibers Containing Silver Nanoparticles
Source: Int J Mol Sci. 2014 Apr 22;15(4):6857–79. doi: 10.3390/ijms15046857 (PMC4013666; doi:10.3390/ijms15046857)
Supplement: Supplementary file 1 [file ijms-15-06857-s001.pdf]

Supplementary Information

Figure S1. UV-vis absorption spectra of silver tetrafluoroborate in acidic solution: (**a**) F100; (**b**) F70W30; (**c**) F50W50; (**d**) F30W70; (**e**) W100.

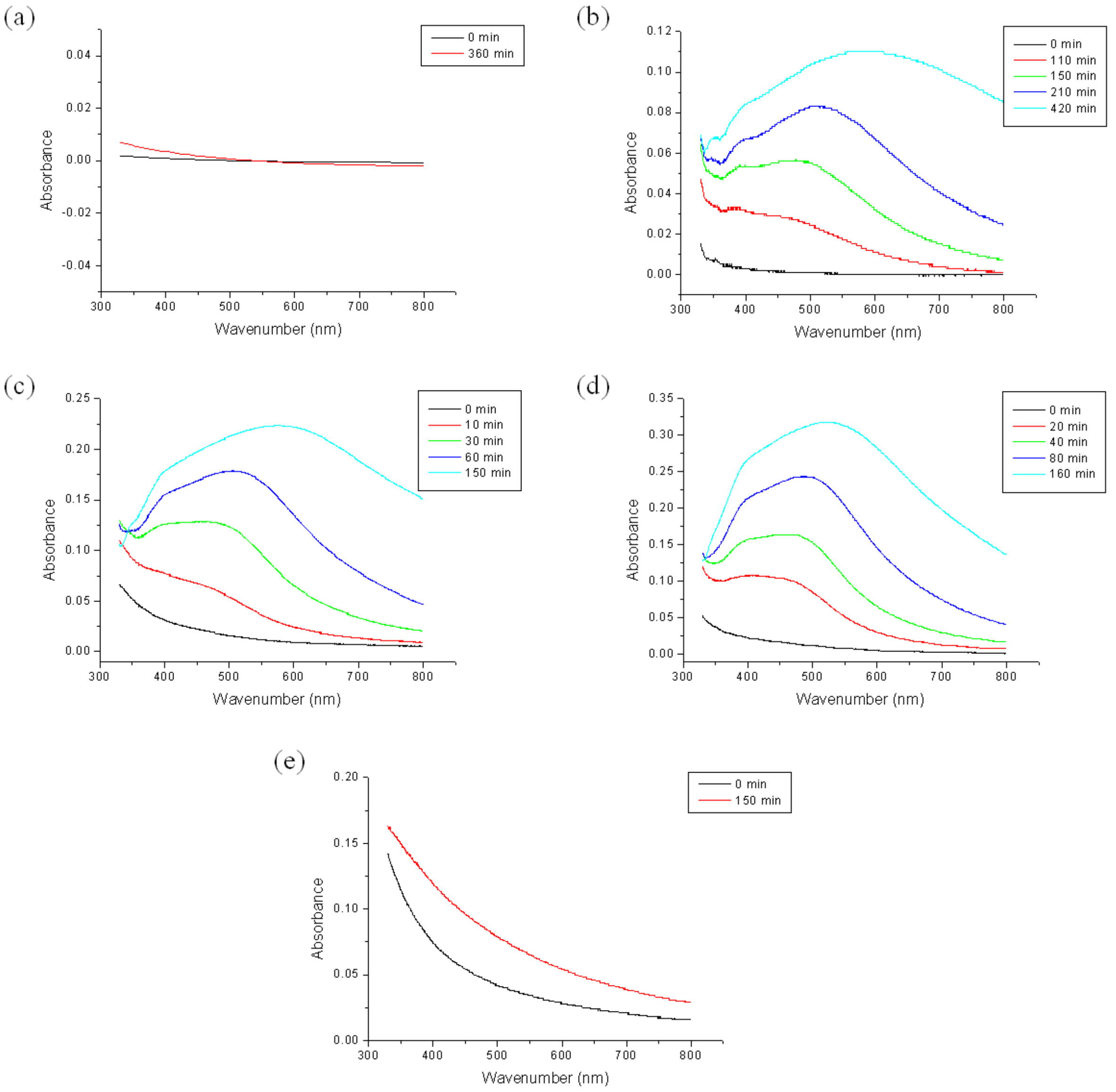

Figure S2. UV-vis absorption spectra of silver phosphate in acidic solution: (**a**) F100; (**b**) F70W30; (**c**) F50W50; (**d**) F30W70.

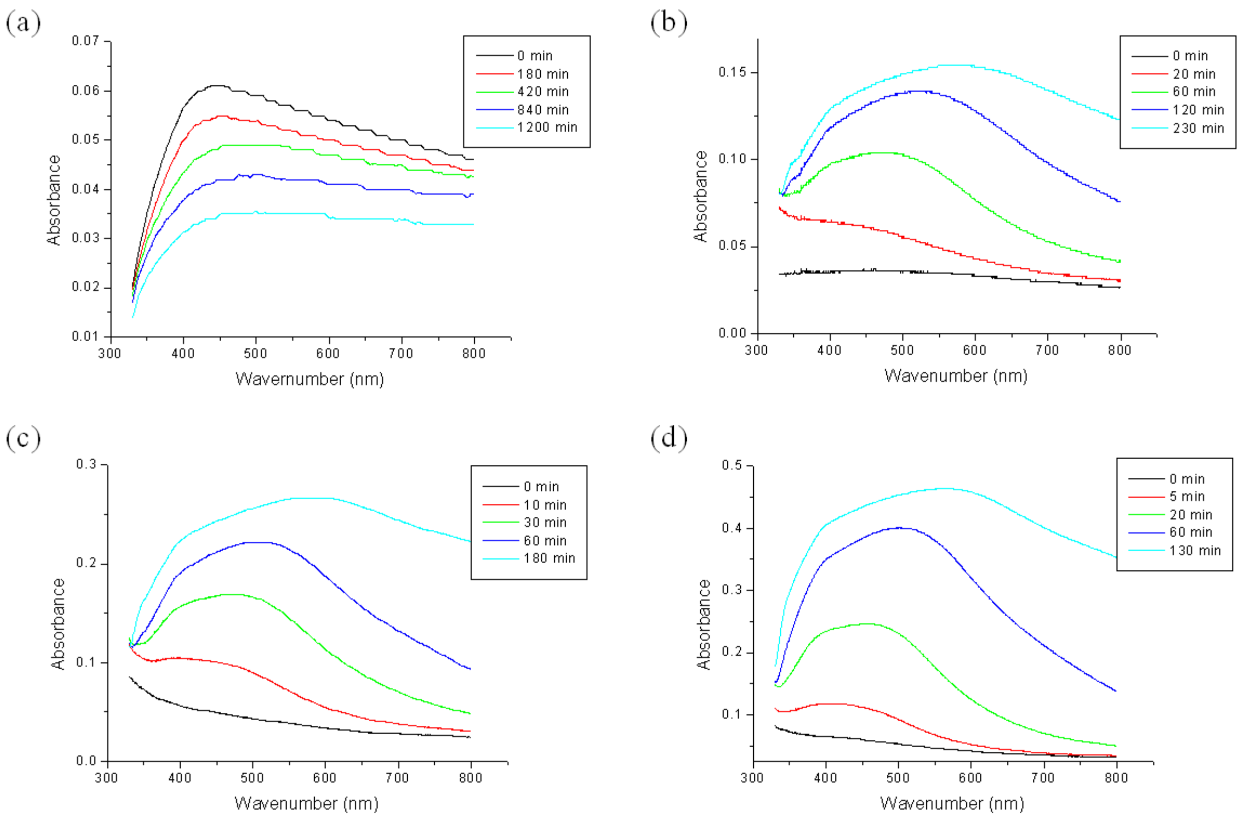

Figure S3. Conductivity of Ag precursor dissolved in acidic solutions: (**a**) silver acetate; (**b**) silver tetrafluoroborate; (**c**) silver nitrate; (**d**) silver phosphate.

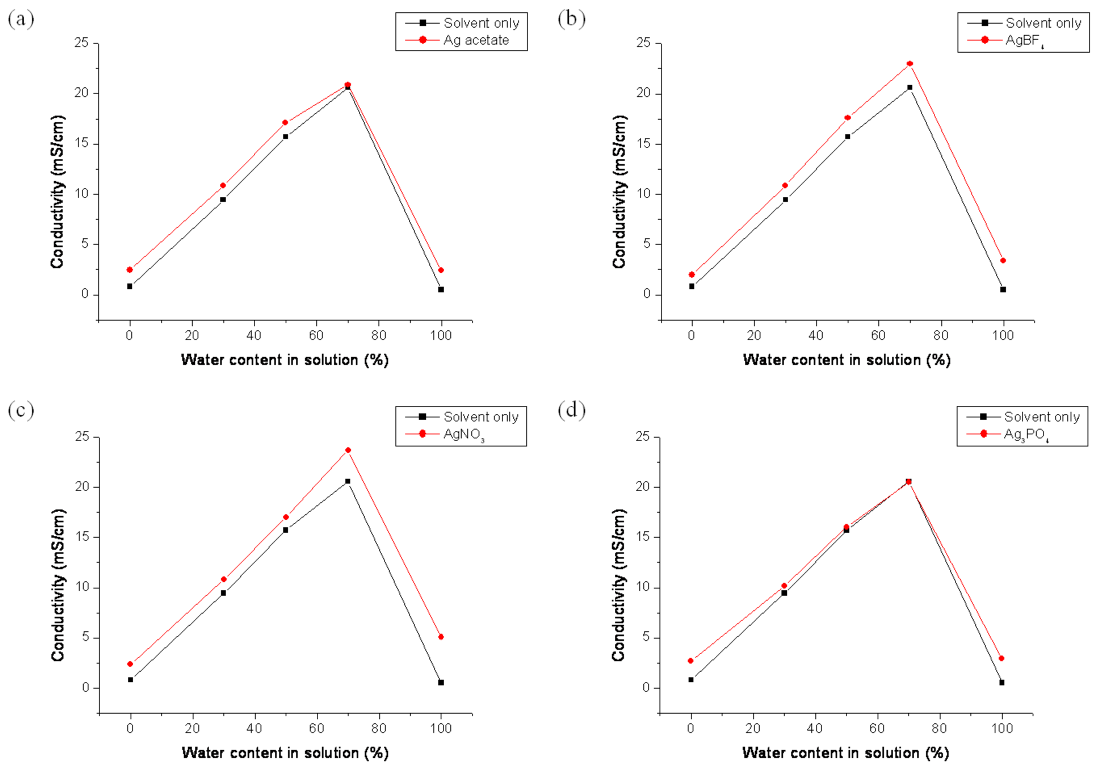

Figure S4. Particle size distributions of Ag nanoparticles in acidic solution: (**a**) F100; (**b**) F70W30; (**c**) F50W50; (**d**) gelatin/F30W70; (**e**) gelatin/F100; (**f**) gelatin/F70W30; (**g**) gelatin/F50W50; (**h**) gelatin/F30W70.

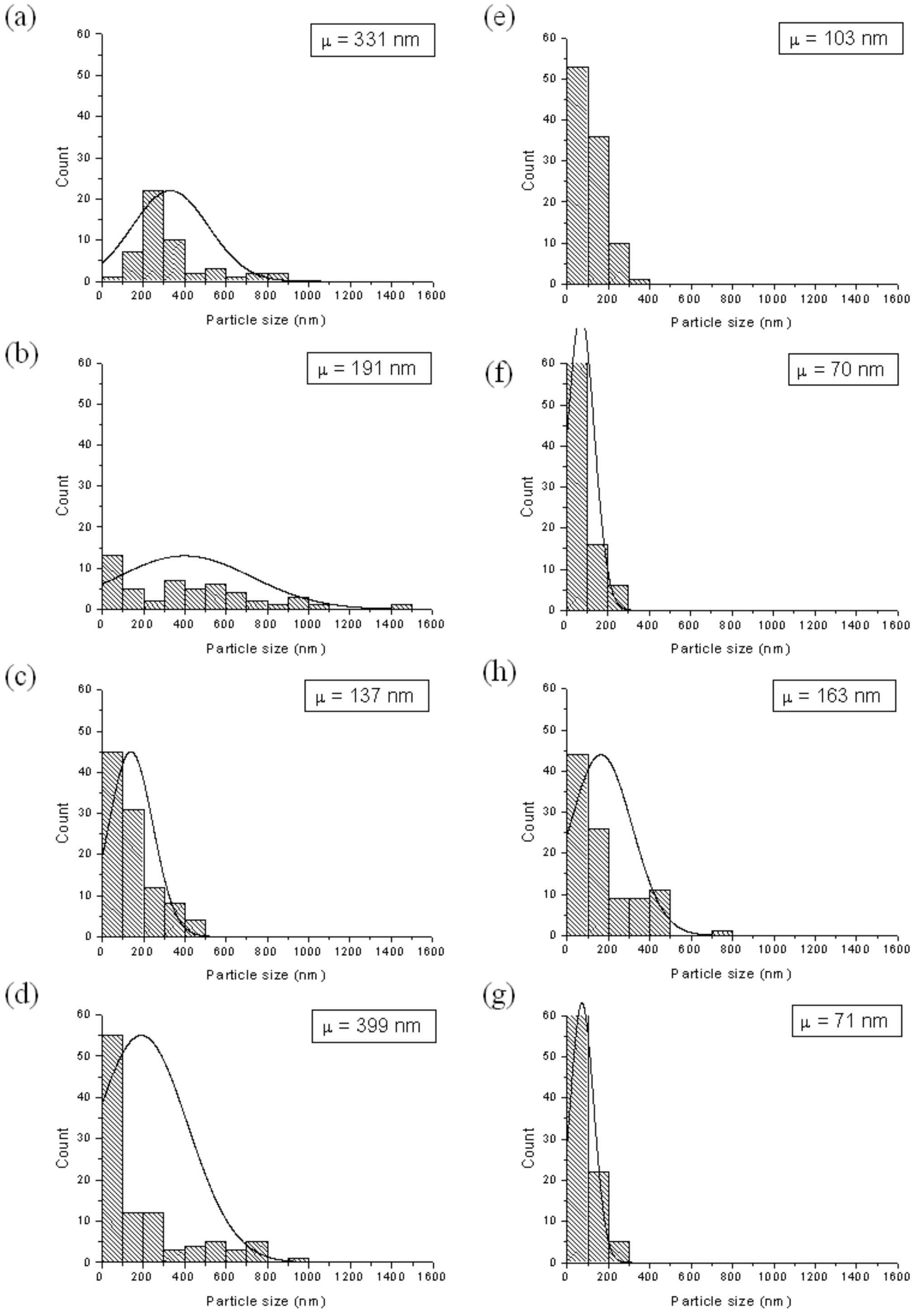

© 2014 by the authors; licensee MDPI, Basel, Switzerland. This article is an open access article distributed under the terms and conditions of the Creative Commons Attribution license (http://creativecommons.org/licenses/by/3.0/).
